# CBCT Analysis of Changes in Dental Occlusion and Temporomandibular Joints before and after MEAW Orthotherapy in Patients with Nonlow Angle of Skeletal Class III

**DOI:** 10.1155/2020/7238263

**Published:** 2020-02-19

**Authors:** Yi Guo, Xinrui Qiao, Shiyu Yao, Tiancheng Li, Nan Jiang, Cheng Peng

**Affiliations:** The Second Hospital of Tianjin Medical University, Tianjin 300000, China

## Abstract

This study focus on the changes of the position and morphology of jaw and condyle after MEAW (the multiloop edgewise arch wire) treatment in adults with a nonlow angle (mean angle or high angle SN − MP > 27°) of skeletal class III (mild to moderate skeletal classs III means −5° < ANB < 0°) malocclusions measured by CBCT (cone beam computed tomography). Twenty adult patients (aged 17-26) with a nonlow angle of skeletal class III malocclusions were selected in this study taken orthodontic treatment by MEAW. CBCT was taken before and after the treatment to analyze the changes of the jaw and condyle. After treatment, the angle of L7-MP decreased 12.2°, L6-MP decreased 10.5°, L1-MP decreased 8.8° (*P* < 0.001 for each) and U1-SN increased (*P* < 0.05). There was no significant changes between anterior and posterior APDI index and between anterior and posterior spaces of the TMJ (temporomandibular joint) (*P* > 0.05). The linear ratio of the TMJ was the LR > 12 before treatment, while it was −12 < LR < 12 after treatment; however, there was no statistically significant difference between them (*P* > 0.05). There was also no significant change in anterior and posterior position and morphology of the condyle within the joint fossa after the treatment by MEAW in this study. MEAW technology in correcting the class III with nonlow angle patients mainly relies on the compensation of distally and posterior mandibular teeth, rather than the mandible and condyle moving backward to establish a neutral occlusal. This study was approved by the institutional ethics committee of the Second Hospital of Tianjin Medical University (No. KYJJ2013002).

## 1. Introduction

Skeletal class III patients with a high angle have always been the Gordian knot of orthodontic treatment. Orthognathic surgery is the usual schedule rationally in patients with severe skeletal class III with a high angle. However, in some mild skeletal discrepancy cases, the patients prefer conservative treatment to improve their facial esthetic and functional concerns [1–3]. To release anterior crossbite of high-angle skeletal class III malocclusion patients mainly through the mandible rotated clockwise, the occlusal plane reversal and tilt compensation of maxillary and mandibular anterior teeth [4]. During the treatment of skeletal class III malocclusion of the late mixed dentition, the condyle point was retrogressively shifted, the mandibular growth was inhibited, the position of the mandibular was moved backward, and clockwise rotation took place with the condyle as the center [5]. The finite element study showed that intermaxillary class III traction would cause the tensile stress on anterior inclined plane and the compressive stress on the posterior inclined plane of the condyle [6]. Because of the stress, condyle appeared to get posterior-superior movement. Some scholars believe that class III intermaxillary traction which is used in the treatment of class III malocclusion may increase the joint area load, causing the condyle to move backward, thereby inducing or aggravating joint symptoms [7]. Literatures report that anterior crossbite could be relieved through orthodontic treatment by means of repositioning the condyle or rotating the mandible. It is generally considered that those common methods have potential risks of causing open bite or TMD [8, 9]. Therefore, the multiloop edgewise arch wire (MEAW) technique developed by Kim provides an effective approach for treatment of skeletal class III malocclusion with high angle [10]. It has not been reported whether the joint load aggravation can be avoided by the multiple L-shaped flexural forces on the corresponding teeth of the multiloop edgewise arch wire (MEAW) orthotherapy. Therefore, the aim of this study was to provide a reliable basis for the orthotherapy of nonlow-angle patients with skeletal class III through the comparison of changes in temporomandibular joints and dental occlusion in patients with nonlow-angle patients before and after MEAW treatment in the radiology.

## 2. Materials and Methods

### 2.1. Subjects and Inclusion Criteria

Twenty patients with nonlow-angle skeletal class III malocclusion were selected as subjects, who visited the Stomatology Department of Second Hospital of Tianjin Medical University during 2014 to 2018. This study was approved by the institutional ethics committee of the Second Hospital of Tianjin Medical University according to criteria of the modified Helsinki Declaration of 1983. All of them had the following characteristics: (1) mesial molar relationship and anterior teeth crossbite; (2) ANB < 0°; (3) mild to moderate skeletal class III (−5° < ANB < 0°); (4) mean angle or high angle SN − MP > 27°; (5) the profile tends to be class III concave type; and (6) symmetrical face, no obvious deviation, no obvious temporomandibular joint symptoms, no history of orthodontic treatment, no history of joint trauma, and no systemic disease. The patients (8 female, 12 male) had an average age of 22.3 years and an age range of 17 to 26 years.

### 2.2. Data Acquisition

The bilateral temporomandibular joint was scanned in the intercuspal position before accepting the orthodontic treatment and after the removal of the appliance by CBCT. All CBCT scans were taken by the same scanner with the same settings (EWOO-VATECH, I tube voltage 90 Kv, tube current 6 mA, FOV: 12^∗^7 cm, Implagraphy Korea Co., Ltd.). All scans were taken with the subject in an upright sitting position with the Frankfort plane parallel to the floor while the median sagittal plane is perpendicular to the ground. The upper and lower teeth were kept at the intercuspal position during the scanning procedure.

### 2.3. Measurement Methods and Items

#### 2.3.1. Cephalometry

**Figure 1:**
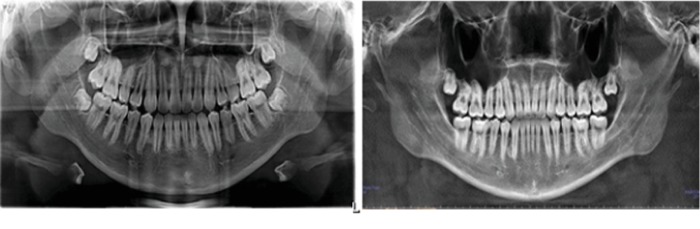
CBCT before and after treatment.

**Figure 2:**
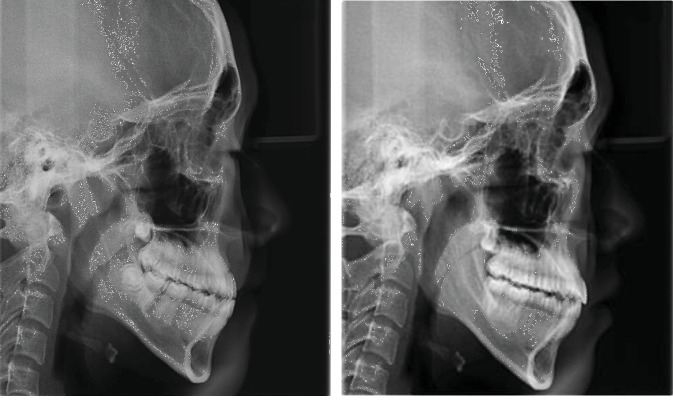
Cephalometric measurements.

CBCT was taken before and after treatment (Figure 1). The sagittal and coronal measurements of the temporomandibular joint and cephalometric measurements of the dental occlusion were performed to evaluate the changes of morphology and location of condyle, and also in teeth (Figure 2).

All the lateral cranial radiographs which were taken before and after correction of those 20 cases were imported into Vcep6.0 cephalic analysis software and define the marking points and measurement items. UMO-6 and UMO-7 were the midpoint of the occlusion of upper first and second molars, respectively; LMO-6 and LMO-7 are the midpoints of the occlusion of lower first and second molars; UMOR-6 and UMOR-7 are the root bifurcation points of the upper first and second molars; LMOR-6 and LMOR-7 are the root bifurcation points of the lower first and second molars.

U7-FH is the angle of posterior and inferior intersection of the long axis of upper second molar (line connecting UMO-7 and UMOR-7) and the FH plane. U6-FH is the angle of posterior and inferior intersection of the long axis of upper first molar (line connecting UMO-6 and UMOR-6) and the FH plane. L7-MP is the angle of posterior and superior intersection of the long axis of lower second molar (line connecting LMO-7 and LOMR-7) and the MP plane. L6-MP is the angle of posterior and superior intersection of the long axis of lower first molar (line connecting LMO-6 to LMOR-6) and the MP plane.

#### 2.3.2. Temporomandibular Joint Space Measurement

Ez3D2009 analysis software was used to perform image reconstruction to obtain images of the sagittal and coronal images.

**Figure 3:**
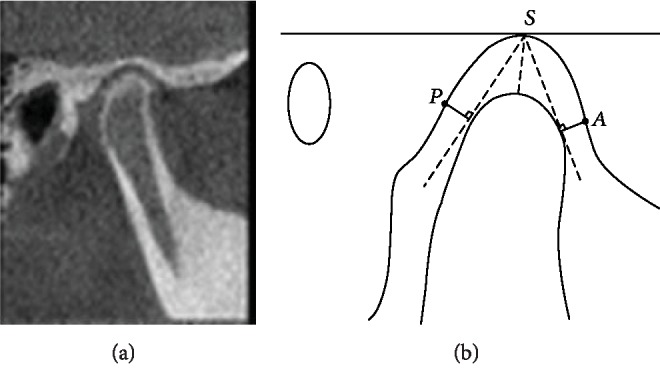
Sagittal measurement. *A*: anterior joint space; *P*: posterior joint space; *S*: superior joint space.

**Figure 4:**
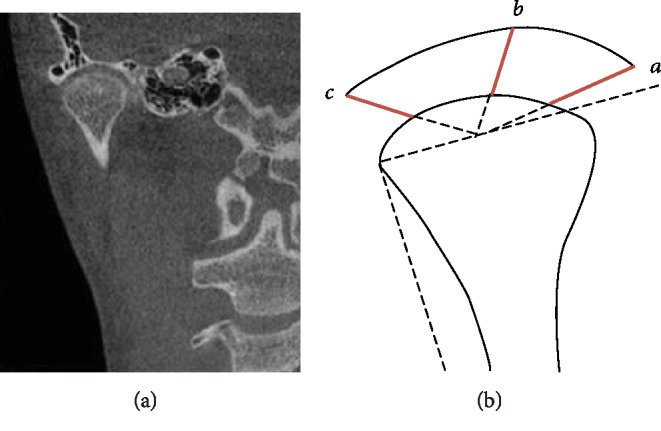
Coronal measurement: A: interior joint space; B: superior joint space; and C lateral joint space.

1. Sagittal Measurement (Figure 3): Anterior joint space (*A*) is the shortest distance between the fossa and the tangency point when tangent to the anterior point of condyle through the top point of the fossa. Posterior joint space (*P*) is the shortest distance between the fossa and the tangency point when tangent to the posterior point of condyle through the top point of the fossa [11]. Superior joint space (*S*) is the distance from the apex of the fossa to the apex of the condyle
2. Coronal Measurement (Figure 4): The line parallel to the FH plane crossing the lateral point of the condyle intersected with the condyle. At the midpoint of the intersection, three lines were connected, respectively, to the innermost point of the articular fossa, the vertex of the articular fossa, and the outermost point. Parts where those lines intersect the condyle and fossa are called interior, superior, and lateral joint spaces

Evaluation of condyle position: The linear ratio (LR) = (P − A)/(P + A)^∗^100 was calculated to determine the location of the condyle. LR<−12 indicates that the condyle moves backward, −12 < LR < 12 indicates that the condyle is situated in the middle of the fossa, and LR > 12 indicates that the condyle moves forward.

**Figure 5:**
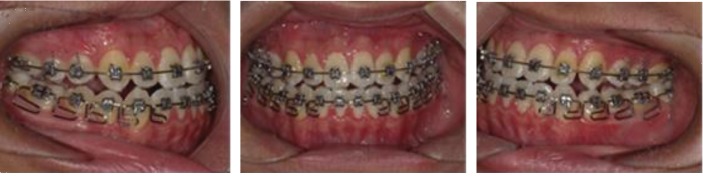
During MEAW treatment.

Orthotherapy method: Lower wisdom teeth were removed in the 20 cases. After regular alignment, in the mandibular, it turned to 0.016^∗^0.022 inches MEAW bow for short class III traction (force value about 100 g) while vertical traction was added to the anterior tooth area for patients with a high angle. MEAW arch was used for both upper and lower jaws of patients with open occlusal tendency. Each L-shaped curve distally tilt force 3-5°.At the same time, patients were requested to hang 3/16 rubber bands for short class III traction for whole and every day (except when eating). The course lasted 13-25.5 months, with an average of 18.6 months (Figure 5).

### 2.4. Statistical Analysis

SPSS19.0 statistical software was used to conduct the paired *t*-test on the measured data between before and after correction (test level bilateral = 0.05).

## 3. Results

After the treatment, all the 20 patients achieved the neutral relationship of molar and the anterior reverse occlusion was relieved. There were no symptoms of temporomandibular joint such as clicking, pain, and limited mouth opening during and after treatment.

Compared with which one before treatment, the angles of the lower first molar L6-MP and of the lower second molar L7-MP were reduced by more than 10° (*P* < 0.001), indicating that both the first and second mandibular molars tilt back toward the distal middle and erect. The lower incisor L1-MP angle decreased (*P* < 0.001), the upper central incisor U1-SN angle increased (*P* < 0.05), the overjet increased about 4 mm, and the difference was significant (*P* < 0.001). Angle ANB increased, but there was no statistical significance (*P* > 0.05). There was no change in the vertical anomaly index ODI and the anteroposterior dysplasia indicator APDI (*P* > 0.05) (Table 1).

There was no significant difference between the anterior and posterior spaces of temporomandibular joints before and after treatment (*P* > 0.05), indicating that the condyles did not have a sagittal shift; the superior joint space was reduced (*P* < 0.05). The linear ratio of joint space LR was 12.8 ± 14.17 before treatment, while it changes to −3.58 ± 9.24 after treatment, indicating that the condyle has a slight shift, but when comparing the change between before and after treatment (*P* > 0.05) (Table 2), we found that this shift is not statistically significant. Coronal and sagittal images showed that the condyle had no hyperplasia and the surface was smooth and continuous, which further indicated that the position and shape of the condyle did not change after MEAW treatment. To clarify the intraobserver reliability, Cohen's kappa of the TMJ space changes was measured and analysed in Table 3.

**Figure 6:**
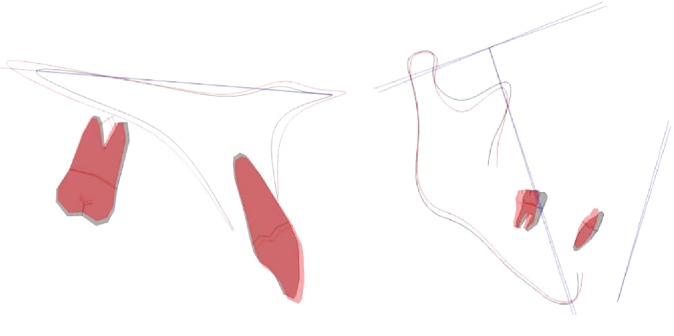
Maxillary and mandibular locus overlap diagrams.

The angles of the lower molar L6-MP and L7-MP both decreased, the distal and posterior tilt were more than 10°, and the lower incisor L1- MP adduction, but the position of the mandible and condyle, did not move (Figure 6).

## 4. Discussion

### 4.1. Mechanism of MEAW in Treatment of Adult with Nonlow Angle of Skeletal Class III Malocclusions

Scholars believe that the condyle plays a key role in maintaining a balanced occlusion and facial outcome [12, 13], and occlusal interference and mandibular dyskinesia caused by malocclusion are the potential risk factors for TMD [14]. The skeletal class III high angle has always been a challenging type for orthodontic treatment. Conventional class III intermaxillary traction may increase the load on joint area. For adult patients who are susceptible to TMD with impacted compensatory ability, posterior condyle movement is a common situation, with the widening anterior joint space [15]. Orthodontic treatment to skeletal class III malocclusion with a high angle sometimes presents poor outcome and leaving the orthodontist in desperation. Open bite or TMD symptoms should be definitely taken into account before the treatment for this kind of cases. This study utilizes the Young H. Kim's Multiloop Edgewise Arch Wire (MEAW) technique to achieve a relatively satisfactory concealment treatment for teeth axis compensatory by adding a distal-tipping force to the L-shaped curve, which is equivalent to a compensation mechanism for patients with abnormal skeletal relationship. The results showed that the L-shaped curved force of the MEAW arch combined with the short type III traction, the angles of the lower molar L6-MP and L7-MP both decreased, the distal-tipped were more than 10°, and the lower incisor L1-MP retraction, anterior cross-bite was corrected; however, there was no significant difference between the APDI and ANB angles which are indicators of jaw sagittal dyssynchrony before and after the treatment. It showed that the treatment did not cause the change of relative position of the maxilla and mandible bones. It is only the compromise of the teeth to relieve the cross-bite of the upper and lower teeth. This method of adjusting the positional relationship of the upper and lower jaws by tilting the vertical molars to establish the occlusion instead of forcing the position of the mandible to retreat can prevent the condyle from moving back to induce TMD symptoms. The space provided by the posterior tilting molar is about 4.5 mm, which is used to retract the lower anterior teeth, in combination with a small amount of flare of the upper anterior teeth, U1-SN is increased, so as to remove the anterior teeth cross-bite and to establish the normal overbite and overjet.

Some scholars have found that increasing of the mandibular plane angle results in the increased long axis tilt angle of the mandibular molars, leaving more upper and lower jaw incisor to compensate, and an enhanced distal inclined the lower jaw molars [16, 17]. This is controversy to the results of Kim et al. that an enhanced mandibular plane angle is always accompanied by more mesial which inclined the molars [18]. However, it suggests that we have a certain limit while using the L-shaped curvature of the MEAW arch to distal erect molars. Therefore, orthodontic-orthognathic surgery is preferred by dentists when treating patients with severe skeletal class III, especially high-angle cases.

### 4.2. The Effect of MEAW on Temporomandibular Joint in Patients with Nonlow Angle of Skeletal Class III

In the orthodontic treatment of skeletal class III malocclusions, the impact of orthodontic force on TMJ have been investigated by many scholars. Nakamura et al. [3]. believed that type III intermaxillary traction would make the condyle retropositioned, increasing the load of joint, and predisposing temporomandibular joint to appear some symptoms such as joint pain, clicking, and jaw lock. But other literatures [16] have shown that two important factors to guarantee successful treatment of adult low-angle skeletal class III malocclusion are the condyle backward movement from the median position and the mandible clockwise rotation. Compared with high-angle skeletal class III cases, the mandibular of the low-angle malocclusion patients were more proven to moving backward, predicting that malocclusion might got accompanied by more obvious dental and functional factors [17]. Instead of moving backward with solely application of class III intermaxillary traction, the condyle could be automatically repositioned backward to the median by means of unlocking the anterior teeth, therefore reliving the TMD symptoms effectively. This theory does not work for patients with a high angle because majority of the cases display a limited mandible bone and condyle making retroposition hard to achieve. Thus, simple class III intermaxillary traction could be utilized to enhance the load force of joint area, pulling back the condyle and leaving the anterior joint space more widen. With the clinical application of miniscrew implants, the field in which cover-up therapy for severe type III skeletal malocclusion may apply turns to be expanded. Al-Mozany et al. [19] suggest that protective orthodontic treatment to the adult patients with high-angle skeletal class III malocclusion could be facilitated by the aid of mini-implants in relieving the class III occlusion by means of moving the entire lower dentition backward.

The bone cortex of condyle tends to be smooth and continuous with less absorption or hyperplasia from CBCT analysis. However, there was no significant change in the anterior and posterior spaces of the joint. The LR is 12.80 before treatment, while -3.58 after treatment, −12 < LR < 12. The result indicate that although the condyle was moved backward to the middle of the articular fossa slightly after the treatment, there was no significant difference in the position of the condyle in the TMJ capsule. The narrower of the superior joint space could be associated with the vertical component in order to resist the clockwise rotation of the mandible and prevent the aggravation of the long-face pattern of growth remains to be explored. He et al. [2, 20, 21] believe that dentists should be cautious when increasing the length of inferior face during the orthodontic treatment of high-angle skeletal class III malocclusion. In cases like that, using of microimplant with a MEAW is an effective alternative method.

In this study, the L-shaped curve of the MEAW arch was chosen to compact with vertical traction in the anterior region; MEAW was used in the upper and lower jaws simultaneously to prevent the extrusion of maxillary molars and the clockwise rotation of mandible when necessary. There was no significant difference in the mandibular plane angle and ODI, which means the risky severe long-face potential factor has been avoided.

Our result was also confirmed by the images of CBCT that this method mainly adjusted the sagittal inconsistent relationship of the jaws with the compensatory tipping of the teeth instead of the backward movement of the condyle, leaving the patient more healthy TMJ circumstance.

## 5. Conclusions

In summary, for patients with mild to moderate nonlow angle of skeletal class III adults, it is safe and effective to use the MEAW technique to correct the malocclusion by dental compensation and may not cause the occurrence of temporomandibular joint disorders.

## Figures and Tables

**Table 1:** Comparison of MEAW measurements before and after treatment in 20 patients.

Project	Before	After	*P*
SNA	79.41 ± 2.57	79.37 ± 3.09	0.959
SNB	81.12 ± 2.93	80.18 ± 2.67	0.079
ANB	−1.72 ± 1.73	0.04 ± 1.57	0.054
U1 to NA	31.30 ± 5.03	34.21 ± 6.06	0.023^a^
L1 to NB	28.15 ± 2.38	19.96 ± 4.00	<0.001
U1 to NA (mm)	8.36 ± 2.05	9.08 ± 3.37	0.455
L1 to NB (mm)	6.79 ± 1.86	4.37 ± 2.43	0.035^a^
U1-SN	110.70 ± 4.20	113.58 ± 3.72	0.015^a^
U7-FH	84.44 ± 9.45	83.27 ± 8.50	0.526
U6-FH	86.62 ± 7.19	91.21 ± 3.60	0.141
L7-MP	89.48 ± 6.53	77.30 ± 4.21	<0.001
L6-MP	86.30 ± 6.06	75.77 ± 5.33	<0.001
L1-MP	94.48 ± 4.25	85.67 ± 3.81	<0.001
*y*-axis	59.19 ± 1.32	59.96 ± 1.42	0.035^a^
OJ (mm)	−0.94 ± 2.18	3.56 ± 0.74	<0.001
OB (mm)	1.27 ± 2.20	1.43 ± 1.00	0.840
MP/SN	32.55 ± 5.21	34.12 ± 4.99	0.052
ODI	58.91 ± 4.11	59.28 ± 5.42	0.699
APDI	93.00 ± 3.22	92.06 ± 4.42	0.613

^a^ *P* < 0.05.

**Table 2:** Changes of temporomandibular joint space before and after treatment in 20 patients.

Project	Before	After	*P*
Sagittal			
Anterior joint space (mm)	2.63 ± 1.11	3.28 ± 1.12	0.140
Posterior joint space (mm)	3.14 ± 0.79	2.90 ± 0.37	0.196
Superior joint space (mm)	3.78 ± 1.00	3.11 ± 0.61	0.016^a^
Degree of condyle displacement (linear ratio)	12.80 ± 14.17	−3.58 ± 9.24	0.051
Coronal			
Interior joint space (mm)	5.61 ± 2.05	6.55 ± 1.86	0.117
Lateral joint space (mm)	4.23 ± 0.87	3.77 ± 0.76	0.061
Superior joint space (mm)	3.80 ± 0.60	3.68 ± 0.63	0.575

^a^ *P* < 0.05.

**Table 3:** Cohen's kappa coefficient calculated by consistency test.

Project	Before (95% CI)	After (95% CI)
Sagittal		
Anterior joint space (mm)	0.49 (0.44-0.54)	0.74 (0.66-0.82)
Posterior joint space (mm)	0.44 (0.26-0.63)	0.56 (0.52-0.60)
Superior joint space (mm)	0.41 (0.27-0.55)	0.56 (0.37-0.75)
Degree of condyle displacement (linear ratio)	0.49 (0.32-0.66)	0.47 (0.32-0.61)
Coronal		
Interior joint space (mm)	0.44 (0.39-0.50)	0.51 (0.34-0.69)
Lateral joint space (mm)	0.38 (0.35-0.40)	0.51 (0.30-0.72)
Superior joint space (mm)	0.49 (0.47-0.51)	0.52 (0.45-0.58)

## Data Availability

The figures and data in the tables used to support the findings of this study are included within the article. This is an open access article distributed under the Creative Commons Attribution License, which permits unrestricted use, distribution, and reproduction in any medium, provided the original work is properly cited.
